# Investigating teaching performance in seminars; a questionnaire study with a multi-level approach

**DOI:** 10.1186/1472-6920-14-203

**Published:** 2014-09-24

**Authors:** Annemarie Spruijt, Jimmie Leppink, Ineke Wolfhagen, Albert Scherpbier, Peter van Beukelen, Debbie Jaarsma

**Affiliations:** Faculty of Veterinary Medicine, Utrecht University, Utrecht, the Netherlands; Faculty of Health, Medicine and Life Sciences, Maastricht University, Maastricht, the Netherlands; University Medical Center Groningen, University of Groningen, Groningen, the Netherlands; Quality Improvement in Veterinary Education, Yalelaan 1, PO Box 80.163, 3508 TD Utrecht, the Netherlands

**Keywords:** Group interaction, Group size, Interactive learning, Seminar teaching performance, Student preparation

## Abstract

**Background:**

Teachers play an important role in seminars as facilitators and content experts. However, contextual factors like students’ preparation, group size, group interaction, and content appear to influence their performance. Understanding the impact of these contextual factors on students’ perception of teaching performance may help to further understand seminar teaching. Besides that, it may help curriculum organisers and teachers to get more insight in how to optimise their versatile role in seminars. The aim of this study is to investigate how students’ perception of teaching performance in seminars is explained by students’ extent of preparation, seminar group size, group interaction, and content.

**Methods:**

The Utrecht Seminar Evaluation (USEME) questionnaire was used to collect information on teaching performance and the aforementioned explanatory variables. To account for intra-student, intra-seminar, and intra-teacher correlation in the data, multilevel regression was used to analyse 988 completed questionnaires in 80 seminars with 36 different teachers.

**Results:**

Group interaction and seminar content had large (*B* = 0.418) and medium (*B* = 0.212) positive effects on perceived teaching performance scores, whereas the effects of students’ preparation (*B* = -0.055) and group size (*B* = -0.130) were small and negative.

**Conclusions:**

This study provides curriculum organisers and teachers indications on how to optimise variables that influence perceived teaching performance in seminars. It is suggested that teachers should search for the most appropriate combination of motivating and challenging content and facilitation method within seminars to optimise discussion opportunities between students.

**Electronic supplementary material:**

The online version of this article (doi:10.1186/1472-6920-14-203) contains supplementary material, which is available to authorized users.

## Background

Interactive group learning is a frequently used educational format in health sciences education, particularly at the undergraduate level [1, 2]. A seminar is an example of an interactive group-learning format defined as "a learning session in which a group of some 25 students discuss questions and issues emerging from assigned readings on a topic of practical relevance and is facilitated by a content expert" [3, 4]. It can be regarded as a constructivist approach to learning. There is a tendency to facilitate a ‘deep learning approach’ when participating in the group prompts asking questions, engaging in discussions and interacting with the subject matter [5, 6]. Students elaborate and restructure facts, principles, and concepts to build robust cognitive frameworks [5, 7, 8] that are assumed to help them apply what they have learned in new situations [9]. Student and teachers stress that the seminar teacher plays an important, versatile role in seminar learning [10, 11]. In a study of Jaarsma et al. [4] the strong relation between teacher performance and the perceived learning effect of students was demonstrated. In the present study, we focus on students’ perceptions of seminar teaching performance to gain a further understanding of seminar teaching and get indications on how to get the most out of these seminar sessions.

Theories on teaching reveal that teaching is a complex activity that is shaped by the teaching context [12]. Teachers in seminars in health sciences education are expected to have good educational skills with regard to questioning, listening, reinforcing, reacting, summarizing, and leadership; they also need to be content experts [10, 11]. According to students, the seminar teacher should possess these skills to be able to stimulate students to actively participate in discussions and promote thinking and problem solving. They should highlight the clinical relevance and enthusiastically guide students toward answers to questions without being threatening [10]. Seminar teachers themselves added that they thought they have an important role in helping students identify gaps in their knowledge, ensuring that learning objectives are reached, and being aware of the placement of the seminar in the course and curriculum so they can estimate the knowledge levels of the students [11]. In short, seminar teaching demands versatile, complex skills and roles of the teachers. These roles are largely comparable to the roles of teachers in groups of 15 students in a hybrid curriculum [13] and to a large extent comparable to the roles of teachers (tutors) in tutorials in a problem-based learning (PBL) curriculum. However, the circumstances in which seminar teachers fulfil their roles are different. Compared to tutors in PBL, seminar teachers facilitate larger groups of students (between 20-30 students per group) and they lack a clear standard procedure (like the 7-jump in PBL) [14] that supports group facilitation.

While it is concluded that a teacher’s performance is not a stable teacher characteristic but affected by the conditions under which he or she works, in existing literature on teaching performance, the influence of the context is often ignored [15, 16]. Gijselaers [17] and Dolmans et al. [18] did investigate the influence of context variables on tutor performance in a PBL-curriculum and showed that the quality of the cases, the structure of PBL courses, the students’ level of prior knowledge, and the level of functioning in tutorial groups influenced tutors’ behaviour in tutorials. In the context of seminar learning, teachers themselves indicated that the students’ extent of preparation, group size, opportunity for group interaction, and the seminar content largely influence how they act in seminars [11]. The relationships between these variables are rather complex because of the many interactions between them. For example, in a qualitative study [10] students explained that the opportunity for and quality of group interaction was a combination of the teacher, the size of the group, the motivation of students, group dynamics, amount and type of seminar questions and the extent of preparation of the students. The extent of preparation depends, among others, on the quality of preparation materials provided by the teacher. We have not found empirical studies that demonstrate the influence of these contextual factors on teaching performance within seminars. According to a qualitative study on seminar learning it is assumed that students’ extent of preparation will be a positive predictor for teaching performance because the seminar teacher is expected to fulfil his roles better with students with more prior knowledge [11]. We also expect that content will be a positive predictor for teaching performance because of the positive associations that were found between the content and teacher performance in an earlier study on seminar learning [4]. It is assumed that students in a seminar with a larger group size will perceive a lower teaching performance because creating a safe learning climate and individual attention and feedback is more difficult for a teacher when the group is larger. We expect that opportunity for group interaction is valued by students and will reflect in teaching performance scores in a positive way based on Spruijt et al. [11].

To be able to get a better understanding of how teachers perform in seminars, it is clear that teaching performance needs to be studied in the context of certain seminar characteristics. There is also a practical reason for conducting this study: it can give teachers indications on how to perform more optimally and effectively within seminars if they have insights in the impact of these variables. Faculty development trainers can use this information to intervene at the individual teacher level and curriculum organisers can benefit from this information to create an optimal context for seminar teachers so that seminars can be as effective as possible.

In this study teaching performance is measured through questionnaires that are filled in by students right after a seminar facilitated by this teacher. For students see a great deal of teaching, they are in an unrivalled position to comment on its performance and quality. Besides that, nonexperts in a subject are uniquely qualified to judge whether the instruction they receive is helping them to learn [19]. Specific theoretical and empirical basis in the work of Ramsden and Entwistle [20] and subsequent studies [21, 22] have shown associations between the quality of student learning and students' perceptions of teaching [19].

In this study we use the USEME instrument for evaluating seminar learning [Spruijt A, Leppink J, Wolfhagen HAP, Bok GJ, Mainhard TM, Scherpbier AJJA, van Beukelen P, Jaarsma ADC: Factors influencing seminar learning and academic achievement, submitted] to clarify how contextual elements in seminar groups may condition students’ perception of teaching performance. By a multilevel study we try to find an answer on the research question: How do the students’ extent of preparation, group size, group interaction, and content explain the perceived teaching performance in seminars?

## Methods

### Educational context

The study was conducted in the first 3 years of the 6-year undergraduate curriculum of the Faculty of Veterinary Medicine, Utrecht University, the Netherlands (FVMU). Approximately 225 new students enroll every academic year. The integrated curriculum of the first 3 years is organised around organ systems (e.g., circulatory system and hepato-biliary system) and focuses on basic science, clinical science, and practical skills. Seminars, lectures, and practicals are the main educational formats. Contact time between students and teachers takes up 30-40% of the total study time, and the remaining time is designated for self-study to prepare for sessions and exams. Assessment consists of written end-of-course exams.

Approximately 40-60% of the contact time is used for seminars that consist of approximately 25 students. The system-based courses comprise approximately eight seminars on different themes (e.g., "Pathophysiology of jaundice and cholestasis"), and nine seminar groups that are facilitated by different teachers are conducted for each theme. Students are expected to prepare for the seminars by reading assignments, mostly with guiding questions. Attending the seminars is optional; usually there is a student attendance of around 75%. With the exceptions of the duration (105 min maximum) and maximum number of students (30), the seminars have no prescribed facilitating method. Every semester (that includes five courses) students are allocated to one permanent seminar group of 25 students by the Office of Educational Affairs. During this semester, these students participate in multiple seminars with the same student group, but teachers vary depending on the seminar theme. The Office of Educational Affairs makes a time-table for every seminar group. Students do not know who will teach the seminar session in advance. Most teachers have attended a 2-year faculty development programme with personal mentoring. Quality assurance is based on student evaluations at the completion of the course.

### Variables

The previously developed instrument for evaluating seminar learning (USEME) [Spruijt A, Leppink J, Wolfhagen HAP, Bok GJ, Mainhard TM, Scherpbier AJJA, van Beukelen P, Jaarsma ADC: Factors influencing seminar learning and academic achievement, submitted] that was based on other studies on seminar learning [4, 10, 11] was used to collect information about the different factors that may explain teaching performance. The underlying items, factor loadings and eigenvalues of the instrument that emerged after principal factor analysis with promax rotation and based on Kaiser’s criterion and scree plot inspection are presented in Additional file 1 as background information. The items consist of statements and students are asked to indicate on a 5-point scale (1 = strongly disagree, 5 = strongly agree). The students’ extent of preparation (measured by three items; *α* = 0.80), content (measured by five items; *α* = 0.82), group interaction (measured by three items; *α* = 0.79), and group size were used as independent (predictive) variables. As the main dependent (outcome) variable, we used perceived seminar teaching performance (measured by eight items related to didactic skills and content expertise; *α* = 0.92).

### Subjects and procedure

Data were collected in samples of all seminars in the second semester of years 1-3 of the undergraduate curriculum at FVMU during April-June 2012. There was an average attendance of 79% of the students during the seminars. All of the participants that attended these seminars were invited to participate in the study. Therefore, in total 1582 questionnaires could have been handed in. The students were asked to complete the questionnaire immediately after the end of the seminar to ensure a proper match between their answers and teaching performance in that specific seminar. After the teacher had left, a student assistant distributed the questionnaire to the students with a letter that explained the aims of the study and stated that participation was voluntary, together with a consent form that requested that the students consent to participate in the study by completing the questionnaire for research purposes. The students were assured that the data would be treated confidentially. The student assistant collected the completed questionnaires and consent forms. She also determined the exact group size for validation. The questionnaires were only analysed if the consent form was signed.

### Analysis

Because individual students are part of the seminar groups and some teachers facilitated multiple seminar groups, multilevel regression was used for the analysis of the questionnaire responses. We used teaching performance scores as the dependent variable and the factors student preparation, content, group interaction, and group size as explanatory (i.e., predictive variables). Multilevel analysis takes interdependence of ratings into account. In the present study, for example, students are nested within seminar groups. Multilevel modelling disentangles these dependencies by quantifying the degree to which variance in student ratings is due to differences between individual students or to the higher level construct that is being evaluated (seminars) and within which ratings are nested. The multilevel method also allows the inclusion of explanatory variables (e.g., seminar group characteristics like group size) that may explain differences between students or between teachers. Another attractive feature of multilevel analysis is that the effects of explanatory variables can be estimated not only as a fixed effect (as in multiple regression analysis) but also as a random effect. This means that it is possible to estimate not only the average effect of, for example, student’s preparation on teaching performance but also the degree to which this effect differs across seminars (random slopes). By choosing multilevel regression instead of multiple regression we are able to deal with the violation of the assumption of independence of observations (students nested in groups, multiple responses of a part of the students) and with deviations from the classical assumption of homogeneity of variances (by specifying random effects). The predictive variables formed the fixed effects of the model. To account for intra-student, intra-seminar, and intra-teacher correlation in the data (i.e., multiple responses from the same students who participated in different seminars, interacting with other students in these seminars, and taught by a smaller number of teachers), we performed the multilevel regression analysis using teacher-level, seminar-level, and student-level intercepts as random effects of our model. P-values of < 0.05 were considered significant. SPSS 21 software was used for the analyses.

### Ethical considerations

Written informed consent was obtained from all of the participants, who were assured that the data would be processed confidentially. The study was approved by the Ethical Review Board of the Dutch Society of Medical Education (NVMO-ERB; dossier no. 46).

## Results

Two questionnaires were excluded because they did not have an attached signed consent form. A total of 988 (62,5%) valid sets of questionnaires, filled in by 410 different students (median number of 2 questionnaires per student, range 1-6), were collected in 80 seminar groups. These groups were guided by 36 different teachers. Twenty of the 36 seminar teachers included in the study guided two or more seminar groups within six different courses.

For student preparation, content, group interaction, and teaching performance, we averaged the scores of items (range: 1-5) within the scale of the factor in question for every student. Table 1 presents descriptive statistics for these four scales and group size.

**Table 1:** **Descriptives of teaching performance scores, student’s preparation, content, group interaction, and group size**

Variable (range)	***N***	Mean (SD)	Skewness	Kurtosis
Teaching performance scores (1-5)	872	4.04 (0.67)	-1.22	2.77
Student’s preparation (1-5)	751	3.48 (0.91)	-0.41	-0.50
Content (1-5)	959	3.97 (0.63)	-0.77	1.20
Group interaction (1-5)	954	3.71 (0.71)	-0.62	0.34
Group size (10-39)	988	20.61 (5.12)	0.99	2.94

The descriptives in Table 1 are shown to give a *conservative* indication of normality because statistics like skewness and kurtosis are based on the assumption of *independent* observations. As explained above, in our study we don’t have independent observations. Unfortunately there are no alternatives for these descriptive statistics for the multilevel analysis method. Because we did not find extreme values or outliers in these descriptives and because of the large size of this study we can interpret that our method seems fairly robust against the deviations of normality that exist in kurtosis. The predictive variables did not have high correlations with each other.

Table 2 presents the outcomes of the aforementioned multilevel regression model. For interpretation purposes, we standardized all of the variables (i.e., mean of zero and standard deviation of one), including the response variable. An advantage of using standardized variables is that the regression coefficients of different predictive variables can be compared in terms of magnitude. A larger coefficient indicates a stronger contribution to the prediction of the response variable.

**Table 2:** **Multilevel linear model for the prediction of (standardized) teaching performance scores**

Parameter	***B***(***SE***)	***df***	***t***-value	***p***-value	Lower	Upper
*Fixed effects*						
Intercept	0.025 (0.073)	31.00	0.345	0.732	-0.123	0.174
Student’s preparation	-0.055 (0.025)	603.93	-2.205	0.028	-0.104	-0.006
Content	0.212 (0.032)	626.62	6.547	< 0.001	0.149	0.276
Group interaction	0.418 (0.032)	620.00	13.225	< 0.001	0.356	0.480
Group size	-0.130 (0.042)	35.11	-3.079	0.004	-0.216	-0.044
	***VAR*** **(** ***SE*** **)**	***df***	**Wald** ***Z***	***p-*** **value**	**Lower**	**Upper**
*Random effects*						
Teacher intercept	0.126 (0.046)	1	2.710	0.007	0.061	0.260
Seminar group intercept	0.041 (0.022)	1	1.841	0.066	0.014	0.118
Student intercept	0.060 (0.025)	1	2.376	0.017	0.262	0.137
Residual	0.256 (0.026)	1	9.841	< 0.001	0.210	0.312

The table shows regression coefficients (B) for fixed effects and variance estimates (VAR) with their associated standard errors (SE) along with *t*-tests and 95% confidence intervals (lower bound and upper bound) based on 751 questionnaires.

Table 2 indicates that group interaction was the strongest predictor of teaching performance scores, and the positive regression coefficient indicates that students who responded with higher scores on group interaction also tended to give better teaching performance scores. The same interpretation appears to hold for content, in which content contributed less to the prediction of teaching performance than group interaction. Group size and student preparation appeared to have a negative relationship with teaching performance. This suggests that the students tended to give lower scores on teaching performance when the group size was larger or when they were more prepared. However, the regression coefficients of these two predictive variables indicate that these two variables have a weaker relationship with teaching performance than group interaction or content.

The larger variance around the random intercept teacher seems to show that there is more variance in this level, but we need to be careful with statements on causality because a part of the random intercept variance can still be explained by other variables that we did not measure in this study. These ‘unmeasured’ variables can be variables on teacher level, but also on seminar or student level.

## Discussion

The present study investigated the extent to which students’ extent of preparation, group size, group interaction, and content are able to explain perceived seminar-teaching performance in order to get a deeper understanding of seminar teaching. It gives curriculum organisers and teachers indications on how to perform more optimally within seminars and enhance the effectiveness of seminars for students’ learning.

The first important finding was that (the opportunity for) group interaction in seminars had a firm positive relationship with perceived teaching performance, indicating that students relate good teaching performance with the ability for group interaction. Our result contradicts the finding of Jaarsma et al. [4] who did not find a significant relationship between the two variables, but underpins what students said in a focus group study on seminar learning [10]. Since empirical work of Ramsden and Entwistle [20] and subsequent studies [21, 22] have shown associations between students' perceptions of teaching and the quality of student learning [19] and multiple studies have shown that group interaction increases the quality of student learning [23, 24], this can be another indication that group interaction within seminars also enhances student learning in seminars. A possible explanation for students relating good teaching performance with the ability for group interaction can be that students in this study have gained insight in the ideas behind seminars because the institution communicates the educational philosophy of the curriculum that states the importance of active learning and group interaction.

A second finding was the negative, although weak, relationship between the students’ extent of preparation and teaching performance. This result appears to indicate that students who spend more time preparing for a seminar value the teachers less. This is against our expectations. Reason for this result can be that some teachers give too much attention to unprepared students during seminars, thus compensating for the students’ lack of preparation by reviewing the preparation materials; therefore, deeper and more elaborate discussions on the subject matter can be missing. This can demotivate well-prepared students and be reflected in teaching performance scores [10, 25]. For a teacher in seminar groups it is hard to deal with many students with different levels of prior knowledge that is due to the variable extent of preparation and the larger group size. One example to prevent these different levels of knowledge might be the introduction of initial prior knowledge assessments before or at the beginning of a seminar (comparable to the Readiness Assurance Test that is used in team-based learning curricula) [26]. Teachers can also decide to approach the less prepared students in another way than the well prepared student. The teacher can discuss the seminar content with a subgroup of well-prepared students while the unprepared students discuss the content with each other and may only consult the other well prepared group of students before they consult the teacher. It is important that seminar teachers are trained in how to deal with these situations to prevent them for taking the role as knowledge transmitter instead of facilitator.

The third finding was the small negative relationship between group size and teaching performance. This relationship appears to indicate that seminars should not consist of too many students. Students may feel that the teacher has little or no time to focus on questions from individual students or is not able to give them useful individual feedback on their activities. Other reasons may be that the students feel less safe asking questions or feel anonymous when they participate in larger seminar classes [4, 10, 27]. This result did not surprise us because there is a general consensus that the optimum size for face-to-face small group meetings is between five and eight [5, 28, 29]. Besides that, for a teacher leading discussions between students it is easier in a small group because she or he can facilitate active participation and knowledge construction of the students better. However, it is stated that groups can function productively and satisfactorily even if their size lies outside those limits, but the facilitator has to work harder and need to show leadership skills more [5, 29]. We therefore think that by dividing the seminar group in subgroups you have can reach ‘best of both ways’.

### Recommendations

The findings of this study indicate the complex role of seminar teachers. Teachers should have skills to facilitate discussion and activate students to promote thinking and problem solving and are dependent on the context they work in. A well performing seminar teacher therefore needs to be able to apply flexible didactical approaches. Some of these context variables are beyond their own control (extent of preparation, group size), but providing the teachers insights in these variables can help them to optimise the variables that are within their power.

What can teachers themselves do to optimise seminars? Teachers are advised to design their seminars in such a way that the content motivates and stimulates students to interact. To be able to stimulate in depth learning the content should go beyond questions. By providing *question*s, there is a risk that we prime our health sciences students that there is only one right question. While in real life, as a doctor or veterinarian, patients are complex and ambiguous. We believe that active group learning methods, as seminars, are good methods to approach the complexity of real-life problems, provided the content holds for challenging *assignments* that are complex and integrate knowledge. These kind of assignments need to demand interaction and verbalisations of the subject matter prepared by students and are assumed to stimulate deep learning [24].

Another recommendation for teachers is to practice with their group facilitation and discussion skills such that interactions between students are optimally facilitated. While we know that the number that optimises interaction and the variety of knowledge, experience, and viewpoints ranges from five to eight people [5], McCrorie [28] states that ‘group size is probably less important than what the group actually does’. Small group techniques can be incorporated into seminars as long as large groups are broken up into smaller units to encourage interaction and active participation. Edmunds and Brown [29] and Dennick and Spencer [5] provided some simple, effective methods for encouraging students to interact and co-construct knowledge by using different facilitating methods (e.g. buzz groups). Faculty development programmes should provide opportunities to practise and experiment with these facilitation methods. We also suggest curriculum organisers not to enlarge seminar group size and to invest in clearly communicating their educational philosophy and aims of the seminar to their teachers and students.

### Strengths and limitations

A strength of this study is that the questionnaires were collected in the *authentic* context of many different seminars. Even though we knew that the method of sampling might bring bias in student response, we believed that the value of the uniqueness and ecologic validity of the results would give us richer insights in the actual teaching performance in seminars. Because the attendance of the seminar groups was on average 79% we think that our results can be interpreted as representative. In future research we can use these insights for more systematic studies on seminar learning, for example by conceptualizing interventions that can be implemented iteratively in seminars. Another strength is the broad variety of seminars and teachers related to the different disciplines and themes we sampled. Consequently, we believe that the results may be valuable to other health professions institutions that use seminars in their curriculum. Although the present study provides rich information about the relationships between factors that explain teaching performance, some limitations exist. The study was restricted to one approach (student perceptions) to examine seminar-teaching performance, although for example Berk [30] indicated that multiple sources of evidence are needed to evaluate teacher effectiveness. Notably, however, students are the ones who should benefit from seminars for their learning and therefore are the main "clients" [19, 27] that can indicate what makes the seminar effective. Another limitation is that the number of times students responded to questionnaires varied to some extent. A stronger sampling design could result in a larger proportion of students responding to the questionnaire in multiple seminars. This could enable researchers to further study within-students and between-students factors contributing to seminar perception and teaching performance. Finally, we cannot make further statements about possible bias in our student population because we do not know the perceptions of students who choose not to participate in the study.

### Suggestions for future research

We recommend investigating the influence of different facilitating methods that teachers use within seminars on outcomes like ‘depth of discussion’, for example in an experimental setup in different groups dealing with the same content. Such an experimental setup will also enable to investigate to what extent the effectiveness of particular facilitating methods is moderated by, for example students’ extent or prior knowledge. We also recommend further research on the critical group size of seminars.

## Conclusions

This study has provided a deeper understanding of the impact of context variables on teaching performance in seminars. Thereby, it gives teachers and curriculum organisers indications on how to get the most out of seminar sessions. Group interaction and content had large and medium positive effects on teaching performance scores, whereas the effects of the students’ extent of preparation and group size were small and negative. We suggest that teachers and course coordinators should search for the most appropriate combination of challenging content and facilitating method within different seminars to optimise discussion opportunities.

## Authors’ information

A. SPRUIJT, DVM, is a Lecturer and PhD candidate, Quality Improvement in Veterinary Education, Faculty of Veterinary Medicine, University of Utrecht

J. LEPPINK, PhD, is a statistician, methodologist, and postdoctoral researcher in education, Faculty of Health, Medicine and Life Sciences, Maastricht University.

H.A.P. WOLFHAGEN, PhD, is an Associate Professor and Educational Psychologist, Faculty of Health, Medicine and Life Sciences, Maastricht University.

A.J.J.A. SCHERPBIER, MD, PhD, is the Dean and a Professor of Quality Improvement in Medical Education, Faculty of Health, Medicine and Life Sciences, Maastricht University.

P. VAN BEUKELEN, DVM, PhD, is a Professor of Quality Improvement in Veterinary Education, Faculty of Veterinary Medicine, University of Utrecht.

A.D.C. JAARSMA, DVM, PhD, is a Professor of Evidence-Based Medical Education & Innovation, University Medical Center Groningen, University of Groningen.

## Electronic supplementary material

Additional file 1: **Psychometric properties of USEME instrument according to Spruijt et al.** (PDF 196 KB)
