# Supplementary material for: Investigating teaching performance in seminars; a questionnaire study with a multi-level approach
Source: BMC Med Educ. 2014 Sep 24;14:203. doi: 10.1186/1472-6920-14-203 (PMC4190460; doi:10.1186/1472-6920-14-203)
Supplement: Supplementary file 1 — Additional file 1: Psychometric properties of USEME instrument according to Spruijt et al. (PDF 196 KB) [file 12909_2014_1033_MOESM1_ESM.pdf]

Additional file 1: Psychometric properties of USEME instrument according to Spruijt et al.

Factors	Items	Rotated factor loadings				Eigenvalues	% of variance
		F1	F2	F3	F4		
F1) Teacher	The teacher explained the topics clearly	**.81**	.13	.01	-.10	6.76	35.6
	The teacher had sufficient expertise on the seminar topic to facilitate the seminar group	**.69**	.06	-.06	-.11		
	The teacher gave useful feedback on my activities	**.66**	-.06	.05	.14		
	The teacher guided us through the questions in an adequate manner	**.82**	.06	.00	-.10		
	The teacher adapted the seminar to students' knowledge levels	**.69**	-.02	.00	.11		
	The teacher stimulated us to use our prior knowledge to answer the questions	**.68**	.04	-.02	.05		
	The teacher created a safe learning environment during the seminar	**.66**	-.11	.01	.02		
	My overall rating of teacher performance is	**.80**	.00	-.00	-.04		
F2) Content	The relevance of the questions for the field of veterinary medicine is sufficiently clear to me	-.16	**.73**	-.08	.06	2.42	12.8
	This seminar was well structured	.17	**.44**	.02	.00		
	This seminar stimulated my interest in the subject matter	.05	**.66**	.01	.05		
	This seminar has increased my understanding of the subject	.14	**.64**	.03	-.05		
	In this seminar it has become clear to me that the content of this seminar is important within the field of veterinary medicine	-.01	**.74**	.04	.04		
F3) Extent of preparation	To what extent did you prepare for this seminar	-.01	-.04	**.75**	-.04	1.68	8.9
	Before the seminar I thought I was prepared sufficiently to be able to participate actively	.01	-.01	**.95**	.02		
	After the seminar I thought I had prepared sufficiently to be able to participate actively	-.02	.04	**.72**	.02		
F4) Group interaction	The questions stimulated group discussion sufficiently	-.07	.20	-.00	**.69**	1.28	6.7
	The structure of the seminar encouraged interaction between students	-.01	-.01	.01	**.82**		
	The teacher stimulated group discussion	.47	-.14	-.04	**.48**		
